# Implementation of entrustable professional activities in emergency medicine: perspectives from clinical instructors

**DOI:** 10.5116/ijme.6876.5de2

**Published:** 2025-07-25

**Authors:** Yi-Fen Wang, Ya-Hui Lee, Yi-Kung Lee, Chen-Wei Lee

**Affiliations:** 1 Department of Senior Citizen Services, National Tainan Junior College of Nursing, Taiwan; 2 Department of Adult and Continuing Education, National Chung Cheng University, Taiwan; 3 Department of Emergency Medicine, Dalin Tzu Chi Hospital, Buddhist Tzu Chi Medical Foundation, Taiwan

**Keywords:** Entrustable professional activity, clinical teaching, emergency clinical instructor, resident

## Abstract

**Objectives:**

To explore how clinical instructors in emergency medicine perceive and integrate Entrustable Professional Activities (EPAs) into their teaching practices, identify challenges they face, and explore the support required for effective implementation.

**Methods:**

This study utilized grounded theory methodology to explore the pedagogical experiences of clinical instructors within emergency medicine. A qualitative approach was adopted, involving semi-structured interviews with participants recruited through purposive and snowball sampling techniques. A total of 18 emergency physicians, each with over five years of clinical teaching experience in a teaching hospital, were included in the study.

**Results:**

The study revealed several critical insights: (a) Emergency medicine clinical instructors are integral in supervising, training, and providing feedback to residents, facilitating their development. (b) The implementation of EPAs is hindered by limited resources, time constraints, and the challenges of delivering real-time feedback. Instructors often experience a lack of confidence in their teaching efficacy, observe diminished motivation among residents, and encounter difficulties in assessing residents' soft skills. (c) The effective implementation of EPAs necessitates a supportive educational environment, a robust reward system, and objective feedback mechanisms. (d) Instructors derive a significant sense of fulfillment from witnessing the progression of residents into competent and autonomous practitioners, underscoring the importance of empowering educators through enhanced training and institutional support.

**Conclusions:**

The findings offer valuable insights for developing of emergency medical education programs and underscore the need for targeted strategies to enhance the quality of clinical instruction in emergency settings.

## Introduction

Competency-Based Medical Education (CBME) represents a significant shift in medical education, emphasizing the use of competency frameworks in the design, implementation, and assessment of training programs.^1^ Entrustable Professional Activities (EPAs) are central to CBME, serving as a mechanism to gauge the trust that supervisors and patients place in learners.^2^^,^^3^ Learners are expected to demonstrate feasibility, observability, accessibility, and reliability in their clinical performance. EPAs enable educators to assess not only the knowledge and skills of learners but also their professional attitudes and behaviors.^4^

Clinical instructors play a pivotal role in medical training. Typically, experienced attending physicians, who have undergone extensive training in teaching theories, techniques, and strategies, conduct medical training. These instructors have adapted to the evolving landscape of medical education, which has transitioned from a curriculum-oriented, instructor-centered approach to a competency-oriented, learner-centered model. EPAs have been instrumental in facilitating this shift. By focusing on problem-solving, immediate applicability, and effective resolution,^5^ EPAs provide clinical instructors with a structured framework to evaluate the trustworthiness of learners. This framework allows instructors to progressively increase the learners’ responsibilities in clinical practice, thereby cultivating their independence and reliability. Moreover, EPAs enable learners to adjust their learning pace according to their individual needs, while also serving as guidelines for clinical supervision.^6^ Training continues until the learner attains the requisite level of clinical competence. Providing constructive and timely feedback is crucial in the implementation of EPAs,^7^ as it allows for individualized evaluations that support learners' progress.^8^ Clinical instructors are tasked with assessing learners’ development, offering feedback, and adapting learning activities or modalities to optimize educational outcomes and ensure the comprehensive development of learners' competencies.

The implementation of EPAs in clinical teaching presents numerous challenges, particularly in emergency departments. Instructors often need to balance their healthcare duties with clinical teaching responsibilities, which is particularly challenging in emergency departments where time constraints are especially demanding. Lee and colleagues^10^ reported that the inherent characteristics of emergency medicine—such as the limited time available for teaching, the energy constraints of attending physicians, and the varying levels of teaching experience among instructors—pose significant obstacles. Without adequate support, instructors may find it difficult to integrate teaching into their already demanding schedules, particularly if they are unprepared for a teaching role.^4^^,^^11^^,^^12^ Increasing the engagement and involvement of clinical instructors in CBME remains challenging, and this difficulty hinders the widespread adoption of CBME principles.^13^^,^^14^ Implementing EPA-oriented clinical faculty training may positively influence the reform of clinical teaching. It is crucial to establish structured training programs and feedback mechanisms, and to encourage clinical instructors to conduct more frequent assessments.^4^^,^^7^ Promoting CBME, refining feedback techniques, and enhancing instructors' confidence in their teaching abilities are essential steps. Instructors should feel a strong sense of responsibility towards their teaching roles.^10^^,^^15^ Diverse assessment data is critical for making informed decisions regarding different EPAs and provides a foundation for evaluating the effectiveness of residents' learning.^16^

The shift to CBME has profound implications for clinical instructors. They must be adept at managing time constraints, integrating EPAs into their curricula, assessing learners' progress, and providing feedback. Taiwan has adopted the CBME approach and has incorporated EPAs into its medical education system. For instance, the emergency medicine curriculum in Taiwan now includes EPAs such as the management of patients with prehospital cardiac arrest, shock, major trauma, poisoning, acute chest pain, acute altered consciousness, and acute respiratory distress.^17^ Although progress has been made in incorporating EPAs into Taiwan's medical education system, further efforts are required to promote their broader adoption and understanding. Current initiatives are still in the preliminary stages^18^^,^^19^ or focus on examining the traits and competencies of clinical instructors.^20^^,^^21^ Exploring the teaching experiences of clinical instructors is essential for the effective implementation of EPA-oriented clinical teaching.

The research questions addressed in this study were: (a) How do clinical instructors in emergency medicine incorporate EPAs into their teaching practices? (b) What challenges do they encounter when implementing EPAs? (c) What resources and support are necessary for the effective integration of EPAs? (d) What motivates these instructors to persist in implementing and integrating EPAs into their teaching practices?

## Methods

### Study Design and Participants

This study employed grounded theory methodology to explore the teaching experiences of clinical instructors in emergency medicine. Grounded theory is an inductive, iterative, interactive, and comparative approach designed to systematically collect, synthesize, analyze, and conceptualize qualitative data.^22^^,^^23^ It emphasizes the rigorous analysis of empirical data to develop concepts that closely represent the underlying essence of the phenomenon under investigation.

Participants were selected from teaching hospitals in Taiwan, where well-established residency training programs are in place. In Taiwan, teaching hospitals provide specialized medical training to residents who subsequently seek employment at the same hospital after completing their training. Purposive sampling was used to select attending physicians from the emergency departments of these hospitals, all of whom had at least 5 years of clinical teaching experience. A total of 18 physicians from 14 teaching hospitals were recruited, all of whom were male. Each participant had between 7 and 16 years of teaching experience and had mentored between 30 and 100 residents.

This study received approval from the Institutional Review Board of Dalin Tzu Chi Hospital. Participants were recruited between January and March 2021 and were informed of the study's objectives, methodology, and confidentiality protocols. Verbal informed consent was obtained from each participant and recorded in audio format.

### Data Collection

Data were collected through semi-structured, in-depth, one-on-one interviews, which provided insights into the participants' teaching experiences.^24^ These interviews aimed to uncover the participants' perspectives and the meanings behind their language and behavior.^25^^,^^26^ Each interview lasted between 40 and 60 minutes, and data collection continued until saturation was achieved. The interviews explored topics such as job responsibilities, teaching challenges, strategies for overcoming difficulties, and positive teaching experiences.

Interview questions were adjusted based on participants' responses to ensure a comprehensive understanding of each participant's viewpoints and emotions.

### Researcher Reflexivity

To maintain objectivity, each interview was meticulously reviewed. Additionally, the data were reviewed by research peers, qualitative research experts, and medical education specialists to verify their accuracy.^27^ The researchers engaged in self-reflection to understand and interpret the data, and team discussions were conducted to clarify the research questions and fully grasp the significance of the findings. This reflexive process enhanced the credibility and rigor of the qualitative data analysis.

### Data Analysis

Data were analyzed using the constant comparative method.^28^ Interview recordings were transcribed and checked for accuracy, and identity-related information was coded to ensure participant privacy. Transcriptions were examined sentence by sentence to explore relationships within and across utterances.^30^^,^^31^ Meaning units were categorized into four hierarchical levels: meaning summaries, units, subthemes, and themes (Table 1).^32^ This systematic analysis facilitated a deep understanding of the data and supported the development of meaningful insights into the teaching experiencesof clinical instructors in emergency medicine.

**Table 1:** Data analysis examples

Theme	Subtheme	Meaning Unit	Summary of Meaning
Challenges in Implementing EPAs	Demanding Workload	Inability toProvideImmediate Feedback	Instructors may be unable to provide timely feedback to residents due to their heavy workload, leading to the potential loss of critical learning opportunities as the rationale behind clinical decisions may be forgotten.
			Allocating sufficient time for feedback is essential, yet challenging, especially in high-volume clinical settings where patient care demands often take precedence.
		Demanding Workload	Teaching responsibilities can only be effectively carried out when the workload is manageable and the instructor is mentally prepared and focused.
			Balancing teaching duties with clinical responsibilities requires significant additional time and cognitive effort, which can contribute to instructor fatigue and reduce the overall quality of teaching.

## Results

This study gathered comprehensive data on the responsibilities, challenges, support systems, and positive experiences of emergency medicine clinical instructors in the context of implementing Entrustable Professional Activities (EPAs).

### Responsibilities of emergency medicine clinical instructors

#### Supervising residents and providing guidance on clinical decision-making

Eight participants emphasized the critical role of guiding and assisting residents by imparting their clinical expertise and recommending suitable interventions.

“Workplace training involves imparting my knowledge and skills to residents and providing clinical guidance. It entails sharing my experiences and guiding residents in performing clinical work.” (Participant No. 13, Male)

“The role of a clinical instructor is to assist residents and provide guidance, suggestions, and so on.” (Participant No. 15, Male)

### Facilitating constructive dialogue with residents and providing formative feedback

Twelve participants highlighted the importance of engaging in meaningful discussions with residents and providing constructive feedback to enhance their clinical skills.

“If I happen to be nearby, I can assist in assessing a patient, and I can provide feedback after the assessment. Typically, residents come over to give a brief report after completing an examination” (Participant No. 11, Male)

“Clinical instructors guide residents, teach, and give feedback. If a resident performs a task differently from how it should ideally be performed, I start a conversation with them.” (Participant No. 14, Male)

### Delivering educational content to enhance residents’ clinical competencies

Seven participants were actively involved in delivering educational curricula through activities such as simulation exercises, case discussions, and journal club meetings.

“I am primarily engaged in facilitating simulated courses for residents. I organize simulation training sessions once or twice a month.” (Participant No. 9, Male)

“I initiate discussions and host writing sessions on medical cases. In the past, there was an extended period (about a decade) where I conducted a monthly journal article discussion within the department!” (Participant No. 10, Male)

### Pedagogical challenges faced by emergency medicine clinical instructors in implementing EPAs

#### Managing time constraints within clinical education

Five participants identified time scarcity as a primary challenge, noting the difficulty of simultaneously observing residents and engaging in in-depth discussions.

“The primary impediment is the lack of time. The topics I aspire to guide students through are exceedingly expansive, and I often don’t have enough time.” (Participant No. 3, Male)

“The foremost challenge is the lack of time. This is an issue with no readily available solution; one must invest time in observation and subsequently engage in extensive discussion.” (Participant No. 5, Male)

#### Balancing clinical responsibilities with the timely delivery of feedback

Three participants reported that high patient volume and demanding clinical duties impeded their ability to provide timely feedback to residents.

“I find it difficult at times to provide feedback. Feedback needs to be given immediately because important details can quickly fade from memory. As time passes, discussion tends to become less intense.” (Participant No 6, Male)

“I cannot teach if I’m too busy. Clinical instructors have to have enough time and be in the right frame of mind to be able to teach while engaging in their clinical duties. This is a predicament.” (Participant No 18, Male)

### Addressing residents’ variability in learning motivation

“Twelve participants expressed concerns about the lack of motivation among some residents, which posed a significant challenge in the teaching process.” (Participant No 15, Male)

“Residents do not always follow your advice—they often believe their own approach is best. This can be disheartening for instructors.” (Participant No 18, Male)

### Navigating uncertainty regarding pedagogical approaches

Four participants indicated that they occasionally doubted the adequacy of their feedback and the alignment of their teaching with established protocols or the latest empirical evidence.

“I would like to get feedback on my teaching methods and to know whether I am imparting accurate skills and knowledge. Instructors should work together.” (Participant No 10, Male)

“Instructors who lack confidence in their approach tend not to speak up. For instance, I often worry that my information on standard treatment protocols is not up to date. Am I familiar with the latest empirical findings or am I merely stating conventional practices?” (Participant No 14, Male)

### Overcoming challenges in the assessment of soft skills

Four participants highlighted the difficulty in evaluating soft skills, acknowledging their importance but also the complexity involved in their assessment.

“Over time, individuals naturally master medical knowledge and skills, but soft skills are complex and difficult to teach. Soft skills can only be cultivated through modeling, establishing paradigms, and engaging in reflective practices.” (Participant No 12, Male)

“Learning outcomes can often be assessed using computer-based simulations and patient feedback; however, soft skills are not easily assessed.” (Participant No 17, Male)

Essential conditions and support for emergency medicine clinical instructors implementing EPAs

#### Cultivating a learning environment conducive to effective teaching

Six participants emphasized the impact of the workplace environment on their teaching effectiveness, underscoring the importance of support from colleagues.

“The entire department should have a shared understanding of the educational objectives of clinical instructors. Otherwise, the instructors alone bear the burden of teaching.” (Participant No 13, Male)

“The cultural milieu within the department affects the effectiveness of teaching. No one should feel disadvantaged by a reduction in patient consultations resulting from educational commitments. A collective agreement on the necessity of teaching is essential.” (Participant No 17, Male)

### Fostering enthusiasm and commitment among emergency medicine clinical instructors

Eleven participants highlighted the necessity of fostering enthusiasm for teaching within the institutional framework.

“Interest in teaching is lacking within the current institutional framework, especially in emergency departments. Instructors need to be compensated for their efforts. Teaching requires significant time, mental exertion, and physical effort.” (Participant No 1, Male)

“Providing feedback and guidance and imparting knowledge to residents take time. When patient caseloads are high, engaging in comprehensive teaching activities is arduous.” (Participant No 11, Male)

### Modeling positive learning behaviors for residents

Six participants emphasized the importance of serving as a positive role model in cultivating residents into effective and engaged learners.

“Clinical instructors are not just mentors; they are role models who must demonstrate effective problem-solving. Their role goes beyond giving advice and feedback; it includes being a positive example of career development.” (Participant No 2, Male)

“Being a positive role model is essential. Residents benefit from having a positive role model. Role models encourage residents to be more diligent.” (Participant No 16, Male)

### Providing comprehensive training programs for clinical instructors

Seven participants stressed the importance of formal teacher training programs to enhance instructional proficiency.

“Formal teacher training is essential. Many clinical instructors have not completed formal teacher training courses. Clinical instructors need comprehensive training to become adept.” (Participant No 1, Male)

“Simply applying previously acquired knowledge is not enough. Platforms or workshops that teach pedagogical theories, methods, and approaches are essential.” (Participant No 9, Male)

### Establishing a structured and objective feedback mechanism for teaching practices

Five participants indicated the need for an objective feedback mechanism, ideally involving third-party input, to assess and improve teaching practices.

“We are uncertain about the effectiveness of our teaching—resident feedback often carries a hint of flattery. A structured feedback mechanism for instructors is lacking, and teaching methods are not being transparently evaluated.” (Participant No 3, Male)

“An objective and systematic mechanism for giving feedback to instructors is lacking. How can one ascertain the effectiveness of their teaching methods? Are mentors available to provide constructive feedback?” (Participant No 16, Male)

### Factors influencing the commitment of emergency medicine clinical instructors to implement EPAs

#### Receiving Positive Feedback from Residents

Five participants discussed the value of receiving positive feedback from residents, which reinforces their commitment to teaching.

“I greatly appreciate receiving direct feedback from residents. One resident expressed gratitude for the assistance I had provided a week earlier, saying that I had helped them effectively manage their patients.” (Participant No 7, Male)

“Some residents take the time to write letters, sharing the details of an entire shift, a specific case, or a teaching activity. I get a sense of accomplishment when I receive written feedback.” (Participant No 8, Male)

### Observing residents’ application of acquired knowledge in clinical practice

Seven participants expressed satisfaction when observing residents successfully apply the knowledge and skills they have been taught in real clinical situations.

“I enjoy seeing residents achieve positive outcomes after effectively applying specific techniques and communication methods” (Participant No 8, Male)

“On one occasion when I was busy, I entrusted a case involving a severely injured patient to a resident. I later commended the resident for their excellent performance in stabilizing the patient’s condition.” (Participant No 12, Male)

### Facilitating the progressive development of resident competencies

Ten participants noted that the guidance provided by instructors plays a significant role in helping residents mature and become independent practitioners.

“Observing residents stand independently after training, capable even in your absence, and seeing tasks flow smoothly without your input fosters a profound sense of accomplishment. True success is when residents no longer require your presence.” (Participant No 2, Male)

“The most gratifying aspect of teaching is the concept of mutual growth. Witnessing residents progress, helping them identify knowledge gaps, and assisting them in overcoming obstacles are the essence of teaching success.” (Participant No 4, Male)

## Discussion

In Taiwan, the increasing emphasis on the implementation of Entrustable Professional Activities (EPAs) within the Competency-Based Medical Education (CBME) framework underscores a national commitment to elevating the quality of healthcare professionals. Clinical teaching in emergency departments is closely intertwined with the urgent nature of emergency care, involving responsibilities such as supervising, guiding, assisting, and advising residents on clinical decision-making and performance.^6^^,^^9^

These responsibilities often include direct observation of residents during patient care, which allows instructors to provide real-time feedback and immediately address any issues or areas for improvement. To further enhance their competencies, residents engage in various educational activities, including simulation training, case discussions, and case documentation.^7^^,^^8^ Feedback, especially following patient care interventions and direct observation, plays a pivotal role in fostering learning, often leading to discussions on specific treatment details or areas for improvement.

Emergency departments, characterized by their complex and high-pressure environments, demand rapid diagnoses and interventions, making the instructional responsibilities of clinical educators particularly challenging. The participants in this study identified several key challenges, including managing time constraints, coping with mental fatigue,^11^^,^^12^ and providing real-time feedback, all of which hinder effective teaching.^4^^,^^10^ In such a fast-paced clinical environment, many residents tend to focus on routine clinical procedures, exhibiting less motivation for exploratory learning. While clinical assessments often emphasize the breadth of knowledge and technical skills, the evaluation of soft skills remains a significant challenge.^16^ Despite their extensive clinical experience, many instructors expressed uncertainty about the effectiveness of their teaching methods, particularly in the absence of systematic pedagogical training.^10^

Nevertheless, a strong enthusiasm for teaching was evident among the study participants. They derived a profound sense of fulfillment from observing the skill development and practical application of knowledge by their residents. This sense of accomplishment fueled their ongoing commitment to education.^5^^,^^6^ The effectiveness of teaching is significantly influenced by the environment in which it takes place. Based on the findings of this study, the following recommendations are proposed: first, fostering a collaborative and positive teaching environment through mutual recognition and participation among supervisors and colleagues is essential; second, developing mechanisms to incentivize teaching is crucial; and third, instructors should actively strive to serve as positive role models for residents.^20^

Enhancing teaching capacity is a critical strategy for advancing clinical education. Clinical instructors should undergo systematic teacher training to enhance their pedagogical skills and their ability to effectively implement EPAs.^3^^,^^15^ Additionally, establishing objective feedback mechanisms is vital for gaining a comprehensive understanding of, and continuously improving, the effectiveness of clinical instruction.^4^^,^^7^

### Strengths and weaknesses

This study was grounded in a robust and comprehensive dataset. The integration of Entrustable Professional Activities (EPAs) into emergency medical departments in Taiwan several years ago has allowed Taiwanese physicians to accrue significant experience in their application. The study participants, each with a minimum of five years of involvement in clinical teaching, brought substantial practical experience to the research. However, the use of purposive sampling as a recruitment method may have introduced selection bias, potentially reflecting the specific interests and predispositions of the participating physicians. Consequently, the generalizability of the study's findings may be limited. Furthermore, the translation of participant responses from Chinese to English could have resulted in subtle shifts in meaning, affecting the interpretation of the data.

### Implications for Future Research

Future research should undertake a comprehensive investigation of the educational needs of clinical instructors in emergency medicine, with EPAs serving as a potential framework for addressing pedagogical challenges. Additionally, future studies should extend the scope of this research by incorporating quantitative methodologies and examining teaching experiences across various medical specialties to enhance the applicability and depth of the findings.

## Conclusion

This study yielded the following key findings: (a) Emergency medicine clinical instructors play a crucial role in supervising residents, directly observing their clinical performance, initiating educational discussions, providing formative feedback, and organizing training programs aimed at enhancing residents' competencies. (b) The primary challenges encountered by these instructors in the implementation of Entrustable Professional Activities (EPAs) include effective time management, the provision of real-time feedback, particularly through direct observation, sustaining confidence in their pedagogical abilities, and the assessment of soft skills. (c) The successful implementation of EPAs necessitates supportive teaching environments, systematic teacher training programs, incentive systems, and the establishment of objective feedback mechanisms. (d) Instructors derive a profound sense of accomplishment from observing residents develop into competent and independent practitioners. These findings offer valuable insights into the field of emergency medicine education, underscoring the need for targeted strategies and resources to elevate the quality of clinical teaching in emergency departments.

### Conflict of Interest

The authors declare that there is no conflict of interest.
